# Reveals of quercetin’s therapeutic effects on oral lichen planus based on network pharmacology approach and experimental validation

**DOI:** 10.1038/s41598-022-04769-z

**Published:** 2022-01-21

**Authors:** Zhibai Zhao, Linglin Wang, Mengna Zhang, Chenyu Zhou, Yanting Wang, Jiangmin Ma, Yuan Fan

**Affiliations:** 1 Dpartment of Oral Mucosal Diseases, The Affiliated Stomatological Hospital of Nanjing Medical University, 136 Hanzhong Road, Nanjing, 210000 Jiangsu China; 2 Jiangsu Province Key Laboratory of Oral Diseases, Nanjing, China; 3 Jiangsu Province Engineering Research Center of Stomatological Translational Medicine, Nanjing, China; 4 Department of General Dentistry, Stomatological Hospital of Xi’an Jiao Tong University, Xi’an, 710004 Shanxi China; 5 Department of Stomatology, Kunshan Hospital of Traditional Chinese Medicine, 189 Chaoyang Road, Kunshan, 215300 Jiangsu China

**Keywords:** Cytokines, Mucosal immunology, Mucositis

## Abstract

Oral lichen planus (OLP) is a localized autoimmune disease of the oral mucosa, with an incidence of up to 2%. Although corticosteroids are the first-line treatment, they cause several adverse effects. Quercetin, a naturally occurring compound, has fewer side-effects and provides long-term benefits. Besides, it has powerful anti‑inflammatory activities. Here, we combined network pharmacology with experimental verification to predict and verify the key targets of quercetin against OLP. First, 66 quercetin-OLP common targets were analyzed from various databases. The protein–protein interaction (PPI) network was constructed. Topology analysis and MCODE cluster analysis of common targets were conducted to identify 12 key targets including TP53, IL-6 and IFN-γ and their connections. Gene functions and key signaling pathways, including reactive oxygen species metabolism, IL-17 pathway and AGE-RAGE pathway, were enriched by Gene Ontology (GO) and Kyoto Encyclopedia of Genes and Genomes (KEGG) analysis. Then, in vitro experiments showed that quercetin interfered with Th1/Th2 balance by acting on IL-6 and IFN-γ to modulate the immune system in treating OLP. Quercetin considerably affected the apoptosis and migration of T lymphocytes in OLP patients. Our study reveals the potential therapeutic targets and signaling pathways of quercetin associated with OLP, and establishes the groundwork for future clinical applications.

## Introduction

Oral lichen planus (OLP) is a chronic inflammatory disease of the oral mucosa mediated by T lymphocytes. It affects 0.1–4% of the population^1^. According to the World Health Organization, OLP is classified as a potentially malignant disorder, and severe complications can result in oral squamous cell carcinoma^2^. There is no complete cure for OLP due to its recalcitrant nature and idiopathic etiology. Therefore, diversified treatment methods are required. Currently, topical corticosteroids are the first-line treatment^3^. Systemic corticosteroids, such as oral prednisone, are often considered for severe, widespread OLP and lichen planus involving other mucocutaneous sites^4^. However, corticosteroids show a greater rate of incidence for adverse reactions, such as transient burning or stinging associated with application, skin rashes, local swelling and osteoporosis^5^.

Immune imbalance has an important role in the pathogenesis of OLP^6,7^, and involves multiple biological processes, signaling pathways and cytokines. Darczuk et al. indicated that free radicals and increased oxidative stress might be involved in the occurrence and development of OLP lesions^8^. Wang et al. confirmed that the mTOR pathway played a vital role in the immunometabolism of T lymphocytes in OLP patients^9^. The HIF1α/PLD2 axis was enriched in OLP lesions, which was thought to be a key regulatory signaling pathway involving T-lymphocyte immunity by promoting T-lymphocyte proliferation and pro-inflammatory phenotype differentiation of OLP^10,11^. Various inflammation-related cytokines from OLP patients, including interleukins (ILs)^12,13^, transforming growth factor-β (TGF-β)^14^, interferon-γ (IFN-γ)^15^ and tumor necrosis factor-α (TNF-α)^16^, have been shown to play an important role in immune disorders. Abnormally expressed cytokines play a central role in the onset and development of OLP^17^. In summary, the complex pathogenesis of OLP complicates its treatment.

Recently, natural plant products have attracted extensive attention owing to their numerous biological properties. Among them, quercetin has been proven to have pharmaceutical value due to its antioxidant, anti-inflammatory, antineoplastic and anti-allergic potential^18^. As a promising compound, it has been widely researched for disease prevention and treatment, including autoimmune diseases and oral inflammatory diseases, such as rheumatoid arthritis^19^, inflammatory bowel diseases^20^, and periodontitis^21^. For example, in experimental animal models, quercetin could considerably decreased the levels of pro-inflammatory cytokines, such as TNF-α, IL-1β, IL-17, and MCP-1, to reduce the inflammatory response in the microenvironment^20–23^. It could also suppress the migration and invasion of corresponding immune cells to reduce the severity of diseases^24^. Interestingly, quercetin or herbal medicine that contains quercetin, has been used in some clinical studies to observe its efficacy. In periodontitis and recurrent aphthous stomatitis, the results indicated that quercetin might relieve pain and promote healing of lesions^25,26^. Moreover, adhesive films, hydrogels and nanoparticles have been developed for drug applications^27,28^ as new topical formulation to ensure the efficacy and safety of quercetin. To date, there has been only one report stating that the pain and severity indices of erosive OLP patients, treated with oral quercetin hydrate capsules, had substantially decreased compared to the control group treated with placebo capsules^29^. Nevertheless, the underlying mechanism of quercetin in OLP currently remains unclear.

**Figure 1:**
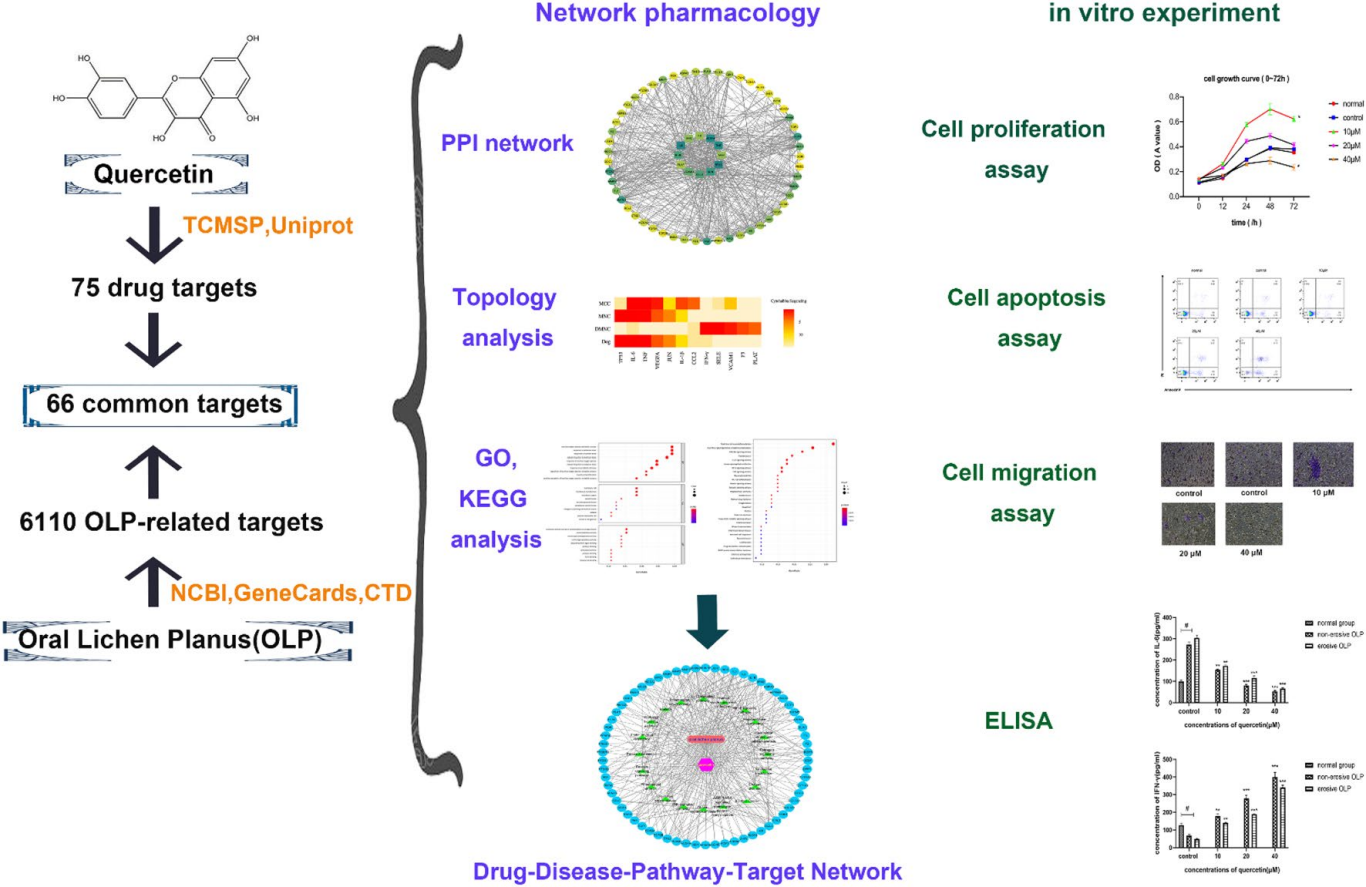
The workflow of this study.

Network pharmacology is a new method of using bioinformatics to observe the interactions between drugs and diseases, providing a new logical guide and technical routes for the development of drugs^30^. Serval studies have used the network pharmacology to identify the mechanism of action of quercetin and systematically elucidate its role in the treatment of diseases, such as rheumatoid arthritis and Alzheimer's disease^31,32^. Unfortunately, there are a few reports on the relationship between quercetin and OLP. Thus, we conducted the present study to identify the targets of quercetin against OLP and analyze the interaction between targets and pathway-related OLP. Further in vitro studies were used to verify our prediction of key immune targets. The workflow of the study is illustrated in Fig. 1.

## Results

### Network pharmacology

#### Acquisition of the common targets of quercetin and OLP

**Figure 2:**
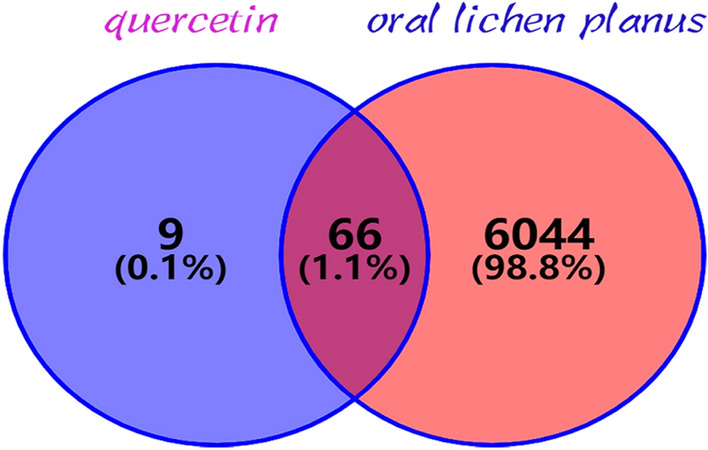
The purple circle on the left represented the targets of quercetin, and the orange circle on the right represented the differentially expressed genes in oral lichen planus (OLP). The overlap part of the two circles represented the common targets of quercetin and OLP.

A total of 75 drug targets and 6110 OLP-related targets were screened from the corresponding database. Subsequently, 66 drug-disease common targets were identified (Fig. 2).

#### Protein–protein interaction (PPI) networks construction, topology analysis and MCODE cluster analysis

**Figure 3:**
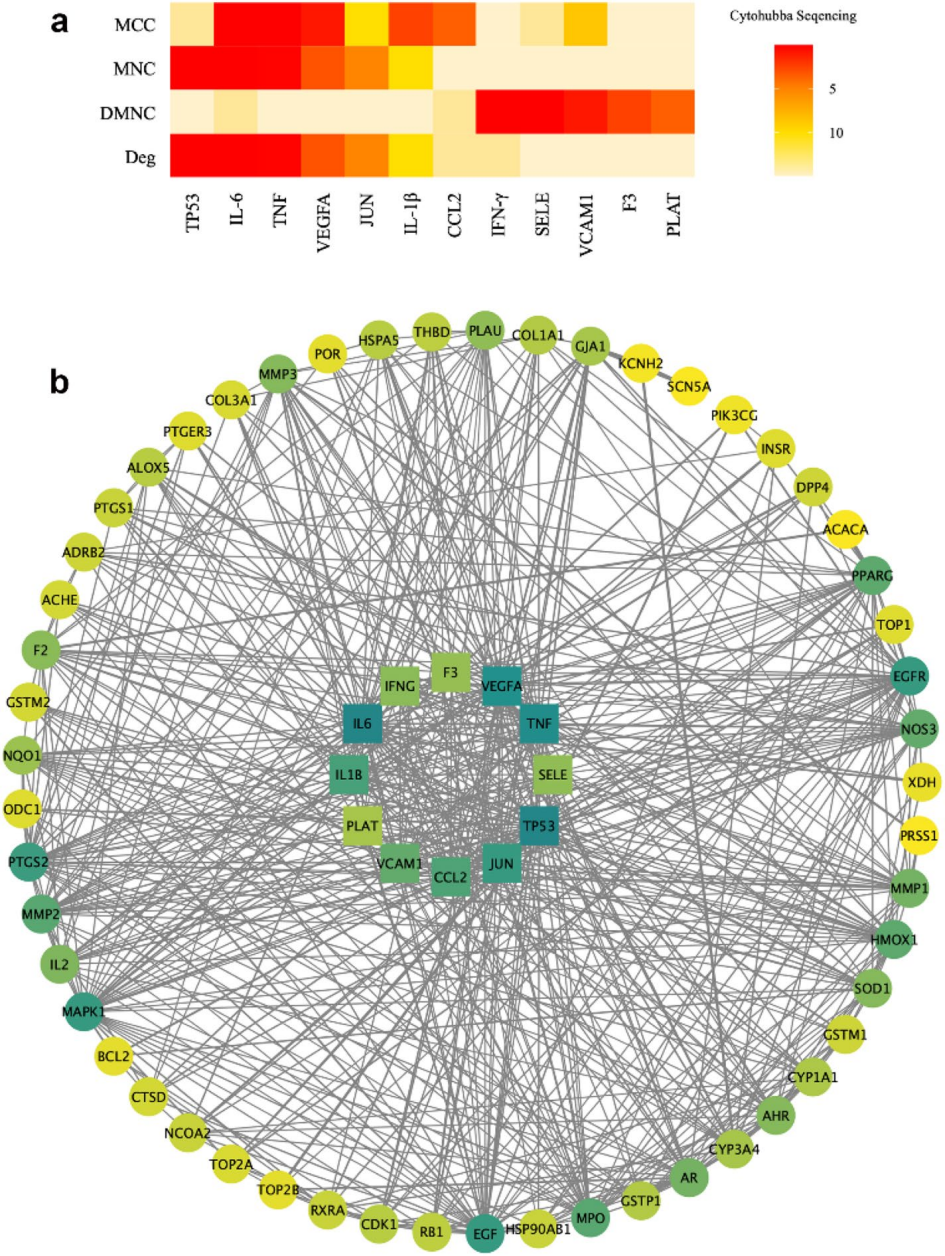
(**a**) Topological analysis was performed by the cytohubba tool. The four algorithms, maximum neighborhood component (MNC), density of maximum neighborhood component (DMNC), maximal clique centrality (MCC) and degree (Deg), were used as reference standards. The darker the color, the higher the sequence. The sequence of each common gene in the four algorithms were integrated. The abscissa represented 12 key common targets. (**b**) This was a protein–protein interaction (PPI) network graph, which showed the interrelationships between the 66 common targets. Each circle represented one common target. The color in the graph was adjusted according to the degree value. The deeper the color, the larger the degree value. The genes in the center of the circle were the 12 key genes that had been calculated using topological analysis.

**Figure 4:**
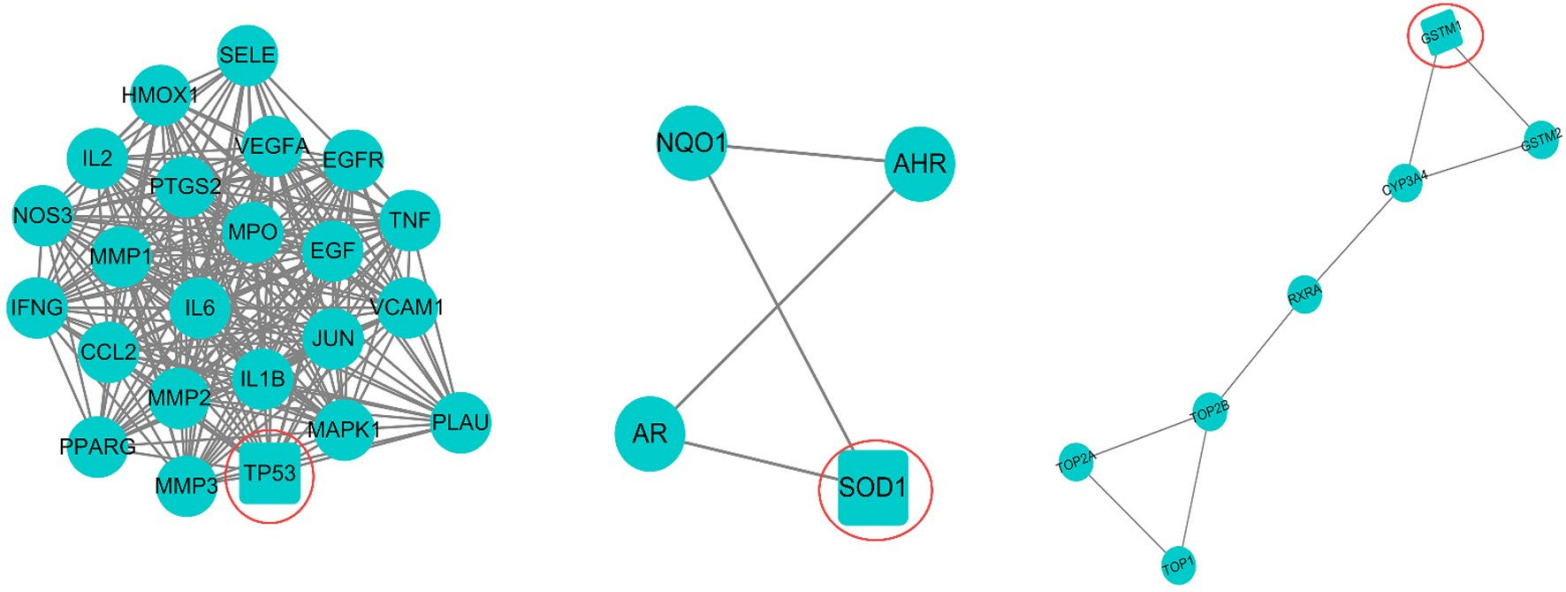
The constructed PPI network was imported into Cytoscape software, and the MCODE module was opened for gene cluster analysis and core target screening. Three gene clusters were obtained. The core gene of each cluster was circled in red.

A protein–protein interaction (PPI) network (Fig. 3b) was constructed to illustrate the relationships between the 66 common target proteins. There were 66 nodes and 609 edges, and the average node degree was 18.5. The color of a node indicated the degree of node contribution in the network. In brief, the 66 common target proteins may react with each other throughout the progression of OLP. Nodes such as TP53, IL-6, and IFN-γ were considerably enriched. Furthermore, 12 key targets were screened by topology analysis (Fig. 3a). The MCODE module was used to obtain three gene clusters, and each cluster had one seed node. The MCODE module is illustrated in Fig. 4.

#### Gene Ontology (GO) and Kyoto Encyclopedia of Genes and Genomes (KEGG) analysis

**Figure 5:**
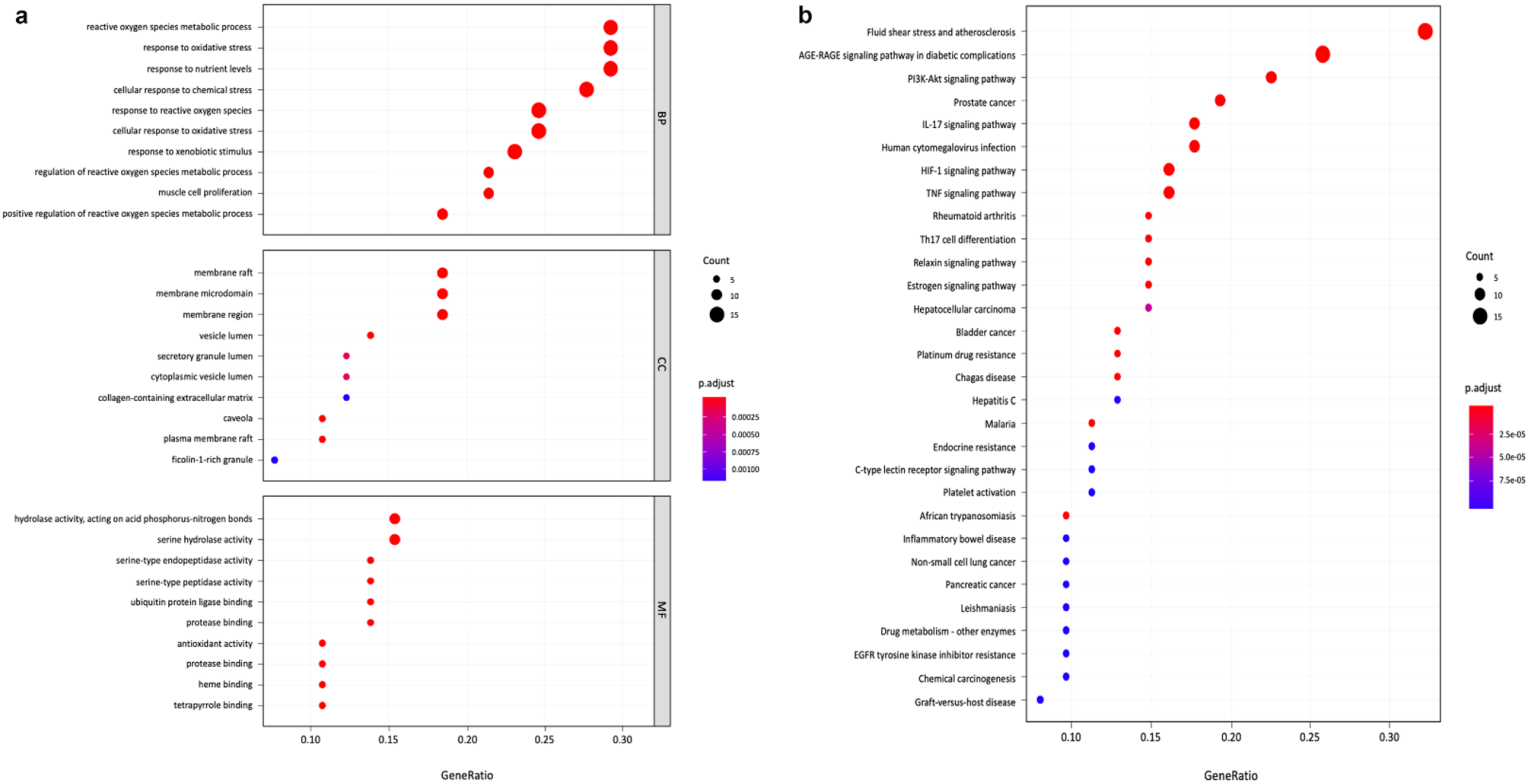
(**a**) The top ten biological process (BP), cell components (CC) and molecular functions (MF) of Gene Ontology (GO) enrichment analysis were listed respectively in order, from top to bottom. (**b**) The common targets were analyzed for Kyoto Encyclopedia of Genes and Genomes (KEGG) pathway enrichment. The importance of top 30 pathways is evaluated and ranked by the bubble diagram.

The 66 common genes targeted by quercetin and OLP were further analyzed for biological processes and KEGG pathways. Among all GO entries, there were 1474 entries related to biological processes (BP), including reactive oxygen species metabolic process, response to oxidative stress, response to nutrient levels and cellular response to chemical stress. A total of 88 items were related to molecular functions (MF), including ubiquitin protein ligase binding, peptide binding, and serine hydrolase activity, and 33 cell component (CC) entries included membrane, membrane microdomain, vesicle lumen, and other similar cell components. Figure 5a shows the top ten items under BP, CC and MF.

To further identify the relationship between target proteins and important pathways, we constructed a target-pathway network from the data extracted from STRING. According to the KEGG analysis, 116 pathways were screened (Fig. 5b shows the top 30), including fluid shear stress and atherosclerosis, AGE-RAGE signaling pathway in diabetic complications, PI3K-Akt signaling pathway, and IL-17 signaling pathway. Logically, the pathways related to as many target proteins as possible were more meaningful than others. These results indicate that quercetin may act against OLP via these signaling pathways.

#### Construction of drug–disease–pathway–target network

**Figure 6:**
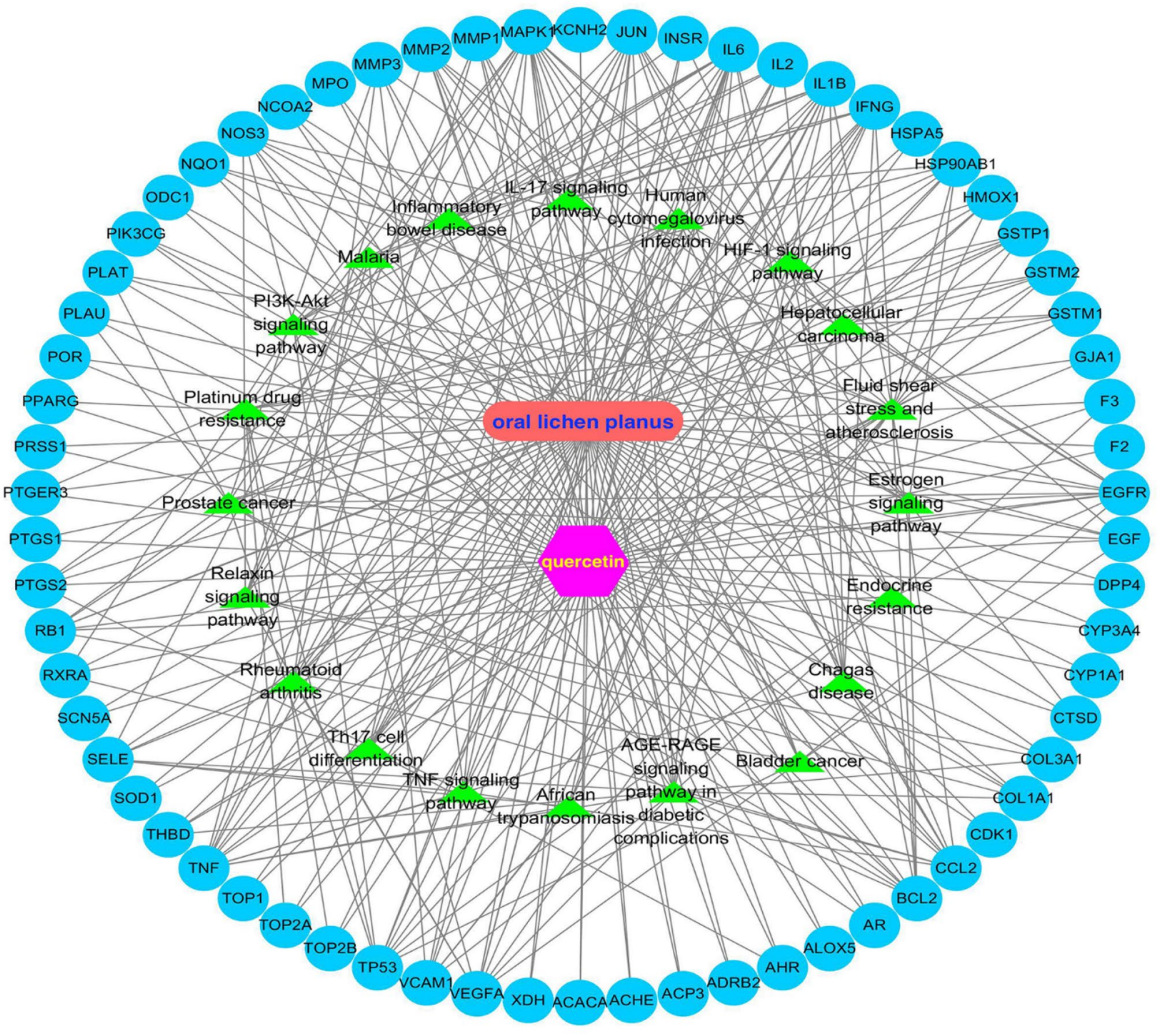
The drug–disease–pathway–target interaction network was drawn by Cytoscape software. The green color indicated the top 20 pathways; the blue circles represented the common targets of quercetin and OLP.

To clarify the relationship between quercetin and OLP directly, a drug–disease–pathway–target interaction network was established, as shown in Fig. 6. The network consists of the drug, the disease, the common targets, and the top 20 pathways, which directly showed the characteristics of multi-component and multi-target effects of quercetin in intervening in the pathogenesis of OLP.

### In vivo experiments

#### Determination of drug concentration

After an incubation with different concentrations (control, 1, 5, 10, 20, 40, and 100 μM) of quercetin for 24 h, the apoptosis rate of T lymphocytes became higher than the control when the concentration was greater than 20 μM (Supplementary Fig. S1). ELISA data showed that IFN-γ protein expression levels (Supplementary Table S2) were significantly decreased, while IL-6 protein expression levels (Supplementary Table S1) were greater than the control when the concentration of quercetin was greater than 10 μM (*p* < 0.05, n = 3) However, there was no considerably difference in the expression of IL-6 and IFN-γ between 40 and 100 μM quercetin. We selected the control, 10, 20, and 40 μM quercetin for further study.

#### The purity of CD3 + T cell

The purity of CD3 + T lymphocytes was 97.31 ± 2.63 (%) in the control group (n = 3), 97.28 ± 0.97 (%) in the non-erosive OLP group (n = 3), and 96.43 ± 0.71 (%) in the erosive OLP group (n = 3). The results showed that CD3 + T lymphocytes in our study were effective.

#### High concentrations of quercetin inhibited proliferation of OLP T lymphocytes

**Figure 7:**
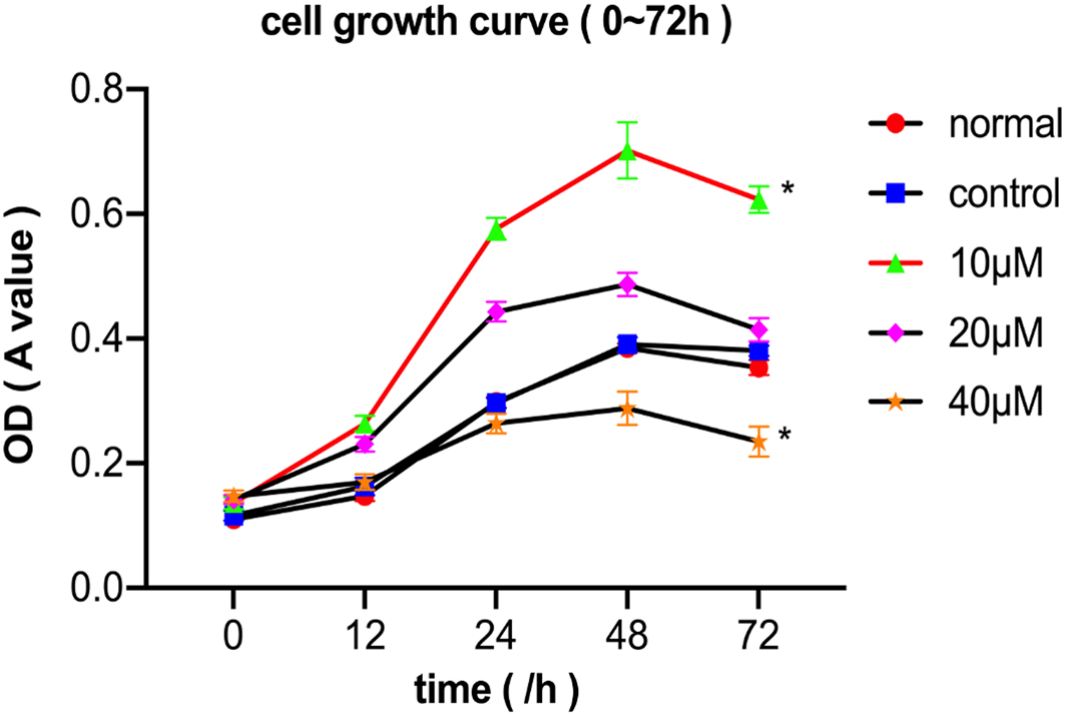
The cell growth curve showed that the 10 μM quercetin group had a proliferative effect on T lymphocytes of OLP patients (the none-erosive and erosive OLP patients, n = 20), while the 40 μM quercetin group showed an inhibitory effect (*p < 0.05 versus the control group). The normal group represented T lymphocytes of healthy controls without any treatments.

Figure 7 shows that cell proliferation in the 10 μM quercetin group was significantly increased compared to that in the other groups in OLP patients and the normal group (*p* < 0.05, n = 20). When the concentration was increased to 40 μM, it showed an inhibitory effect.

#### High concentrations of quercetin induced apoptosis of OLP T lymphocytes

**Figure 8:**
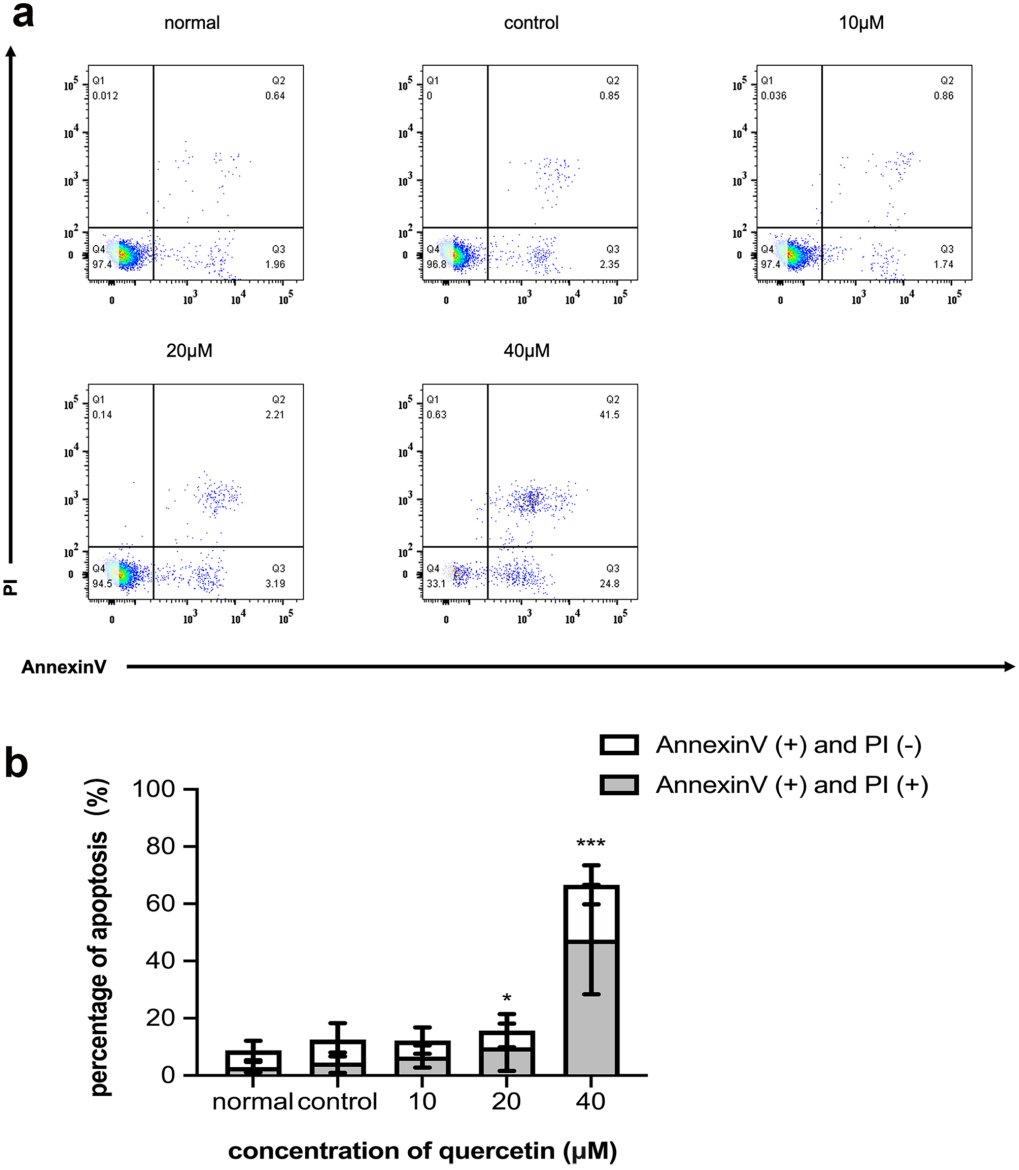
(**a**) Dot plot showed percentage (%) of early apoptotic (right lower quadrate) and late apoptotic cells (right upper quadrate). (**b**) The rate of apoptosis was composed of early and late apoptotic rate. The 20 and 40 μM quercetin group both had a high apoptosis rate (*p < 0.05, ***p < 0.001 versus the control group, n = 20) which was analyzed by ANOVA test.

As shown in Fig. 8a,b, the levels of apoptotic cells in the 40 μM quercetin group were significantly greater than those in the other groups after incubation for 24 h (*p* < 0.05, n = 20).

#### Different concentrations of quercetin on the migration of OLP T lymphocytes

**Figure 9:**
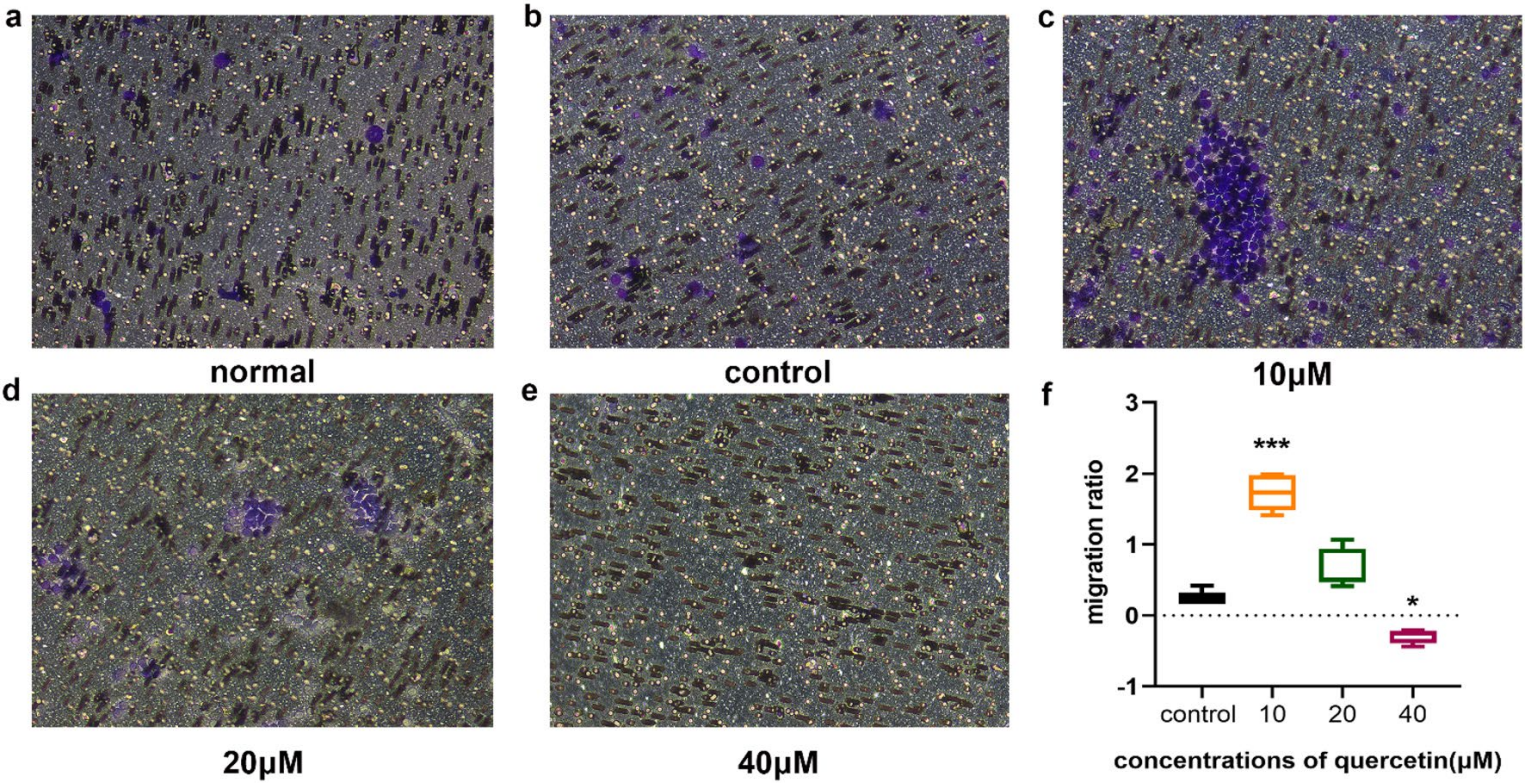
Transwell migration assay was performed to determine the effect of quercetin on T lymphocytes migration. (**a**) The migration of the normal group without the use of quercetin. (**b**–**e**) The migration of OLP patients with different concentrations of quercetin (control, 10 μM, 20 μM, and 40 μM) respectively. (**f**) The analyzed data indicated that 10 μM quercetin had a positive effect on the migration of OLP T lymphocytes. However, 40 μM quercetin had a significant inhibitory effect on the migration of T lymphocytes (*p < 0.05, ***p < 0.001 versus the control group, n = 20).

Incubation with 10 μM quercetin for 24 h had a positive effect on the migration of OLP T lymphocytes (*p* < 0.05, n = 20), and the clustering of cells was obvious. When the concentration of quercetin was increased to 20 μM, the number of cells that migrated out was considerably reduced. In addition, there was no obvious positive migration effect when the concentration of quercetin reached 40 μM (Fig. 9).

#### Quercetin increased the level of IFN-γ and decreased the level of IL-6 of OLP T lymphocytes

**Figure 10:**
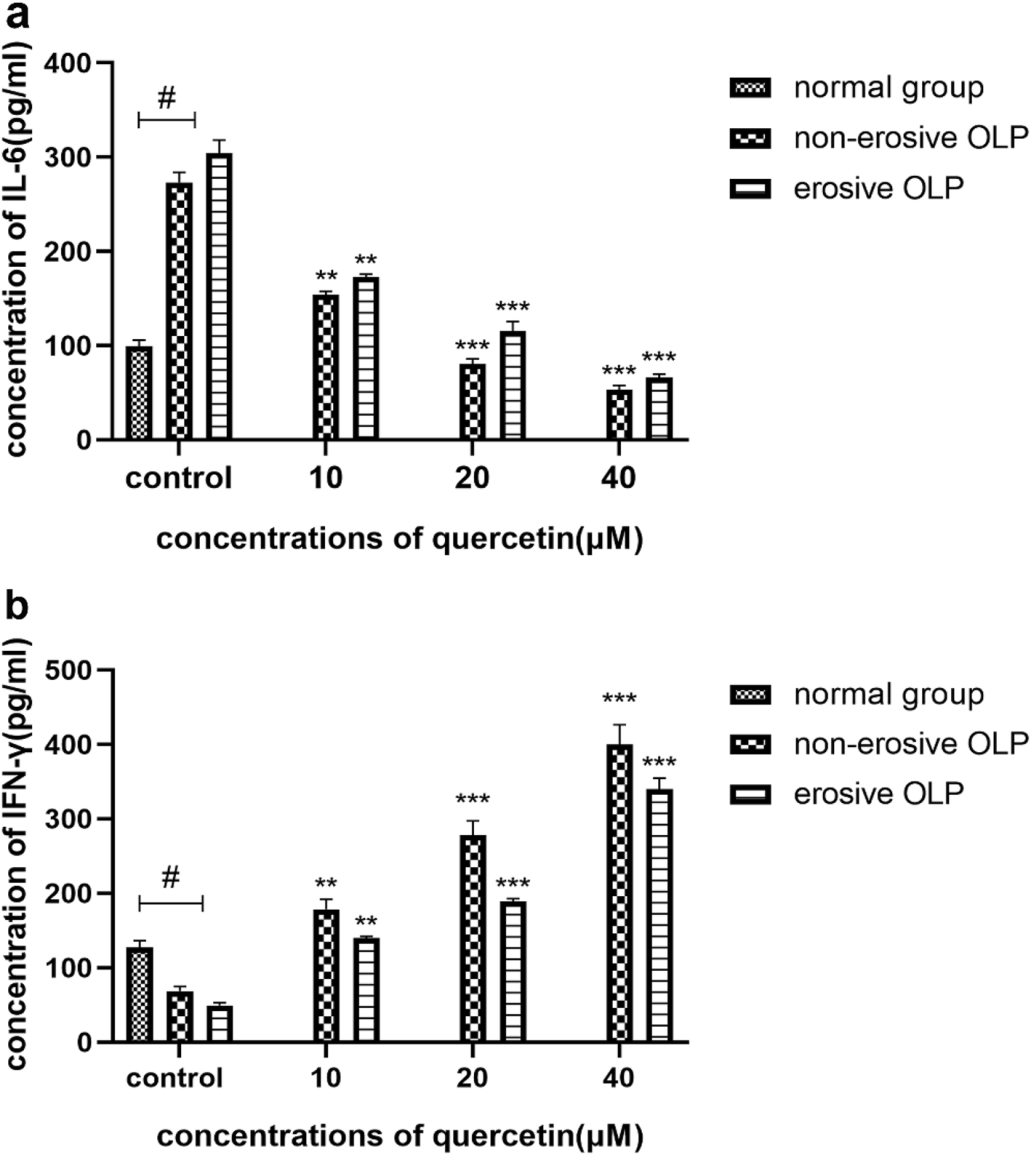
After the incubation with different concentrations of quercetin for 24 h, the expression level of IL-6 (**a**) and IFN-γ (**b**) in the T lymphocytes culture supernatant of the none-erosive and erosive OLP patients was assessed by ELISA. (**a**) showed that the 10, 20, and 40 μM quercetin group had a considerably lower expression of IL-6 in the patients with OLP than the control group. The normal group had lower expression of IL-6 than OLP patients. (**b**) Meanwhile, the 10, 20, and 40 μM quercetin group induced a significantly higher IFN-γ protein level in the patients with OLP than the control group. The normal group had higher expression of IFN-γ than OLP patients. (**p < 0.01, ***p < 0.001 versus the control group of OLP, ^#^*p* < 0.05 versus OLP group, n = 20).

ELISA data revealed that IFN-γ protein expression levels were significantly decreased in the T lymphocytes of the non-erosive OLP group and the erosive OLP group (*p* < 0.05, n = 20), while IL-6 protein expression levels were greater than those in the normal group (*p* < 0.05, n = 20). Considerably, with the increase of quercetin’s concentration, there was an increased level of IFN-γ and decreased level of IL-6 (Fig. 10).

## Discussion

Although the etiopathology of OLP is still unknown, substantial evidence has supported a central role for immune dysregulation in the pathogenesis of OLP^8,10,17^. Various treatments have been applied to treat OLP, but complete resolution is difficult to achieve. Topical corticosteroids are the first-line therapy^3^. However, corticosteroids show a high incidence of adverse effects, such as skin rashes, osteoporosis and gastrointestinal upsets^5^. Quercetin, a natural substance, has a wide range of biological activities, including anti-carcinogenic, anti-inflammatory and antiviral activities^33^. Both in vivo and in vitro studies have highlighted its effect in the treatment of oral cancer^34^, periodontitis^25^, and caries^35^.

In our study, we first identified 66 common targets of quercetin and OLP. Furthermore, three interrelated gene clusters, three core genes (TP53, GSTM1, and SOD1) of the gene clusters, and 12 key genes, including IL-6 and IFN-γ, were constructed. GO and KEGG enrichment analyses revealed that reactive oxygen metabolism response, reactive oxygen stimulus response, cytochemical stress response, and other biological processes and pathways which include AGE-RAGE, IL-17, and HIF-1 pathways may be involved in the intervention of quercetin in OLP. Considering the above results, network pharmacology implies that this is a multi-channel approach for quercetin acting on OLP. Most studies have shown an increased expression of TP53 in OLP lesions, which may be related to the possible malignant transformation of OLP^36^. Studies have shown that a considerably increase in the level of IL-6 and decreased levels of IFN-γ in patients with OLP, thus modulating Th1 and Th2 immune responses and contributing to the immune dysregulation of OLP^3,37^. Mazzarella et al. showed that OLP patients had greater levels of MMP mRNA expression, and MMP1 may be principally associated with erosion development^38^. Lu et al. found that keratinocyte-derived IL-23 might contribute to the accumulation of Th17 cells and the overproduction of IL-17 in local lesions of OLP. IL-17 selectively induced the keratinocytes to produce numerous inflammatory mediators, which resulted in a complex immune network in the inflammatory environment of OLP lesions^12^. High expression levels of HIF-1α in OLP lesions were also reported to be involved in the upregulation of glycolysis and chronicity of oral mucosa lesions^10,11^. All evidence indicates that quercetin may interfere with the development of OLP through multiple signaling pathways and biological processes. Furthermore, the possible mechanisms of action of quercetin on OLP are not only the direct action on multiple targets, but also the interaction between the targets. This is probably a result of different targets existing in different drug action processes and disease progression. We cannot ignore that the method of drug therapy is more than simply up-regulating or down-regulating one or more genes respectively. Drugs with multi-target regulatory functions may be more suitable for diseases with complex pathogenesis, providing new hope for therapy.

Based on the results of network pharmacology, we further studied the effect of quercetin on OLP in vitro. Many studies have claimed that T cell-mediated immune responses play a key role in the pathogenesis of OLP^39^. The proliferation, differentiation, and apoptosis of activated T lymphocytes in OLP are essential for the stability of the immune system^40^. Our study demonstrated that different concentrations of quercetin had a two-way effect on proliferation, apoptosis and migration of T lymphocytes in OLP patients. When the concentration of quercetin was less than 20 μM, it promoted the proliferation and migration of T lymphocytes in patients with OLP. In contrast, 40 μM quercetin had a significant apoptotic effect. At the same time, there was a significant increase in the level of IFN-γ and a decrease in the level of IL-6 with increasing quercetin concentration. Our previous studies have found that Th2 cells may play a leading role in the Th1/Th2 immune balance and the pathogenesis of OLP in the immune environment of the peripheral blood of patients^41^. To date, growing evidence indicates that the Th1/Th2 balance in the cytokine network may greatly influence OLP immunopathology. Investigations of Th1 cytokines in OLP showed that serum IFN-γ and IL-2 levels were less in healthy controls, while the expression of pro-inflammatory Th2 cytokines IL-6 and IL-4 was elevated^42,43^. The Th1/Th2 drift in OLP patients may be closely related to the prolonged course and difficulty in curing the disease^44,45^. Thus, the use of quercetin can correct the immune imbalance by regulating the secretion of Th1 and Th2 cytokines, which is expected to be a new treatment method for OLP. Many reports have demonstrated that decrease in the viability and migration of T lymphocytes, and increase in apoptosis after quercetin treatment is beneficial in the management of inflammatory diseases and cancer^31,46,47^. Based on our findings, we propose that high concentrations of quercetin inhibit cell proliferation and migration, and induce T lymphocyte apoptosis in OLP patients, which may explain the immunomodulatory effect of quercetin against OLP.

The combination of the network pharmacology method and in vitro experiments made our research more reliable and clarified the mechanism of quercetin in treating OLP. However, this study has a few limitations. Firstly, network pharmacology is an analytical method that based on large data sets^48^. If certain targets are not reported in a high proportion, the critical pathways involved in the progression of OLP may remain undetected. Secondly, other key targets, signaling pathways and biological reaction processes remain unverified. For future research, RNA sequencing can be applied to compare gene expression, which can detect multiple targets at the same time and make the results more sensitive and accurate. In addition to the in vitro experiments, in vivo experiments should be performed.

## Conclusion

In summary, based on the network pharmacology, multi-targets and multi-pathways that have a role in OLP progression were used to explain the therapeutic effect of quercetin. This current work is the first documented research to propose this mechanism. Correspondingly, in vitro experiments showed that 40 μM quercetin inhibited the proliferation and migration of T lymphocytes in the OLP immune microenvironment. It also led to a considerably increase in the level of IFN-γ and decreased level of IL-6, which interfered with the Th1/Th2 balance to modulate the immune system. Our results provid a novel way to explore the therapeutic effects of quercetin on OLP.

## Methods

### Network pharmacology

#### Potential targets of quercetin

Quercetin targets were obtained from the Traditional Chinese Medicine Systems Pharmacology Database and Analysis Platform (TCMSP, http: //tcmspw. com), and “quercetin” was used as a keyword. Furthermore, the genes corresponding to the targets were searched using the UniProt database (https://www.uniprot.org).

#### Screening the differentially expressed genes in OLP

The differentially expressed genes in OLP were screened using the following three databases: GeneCards (https://www.genecards.org/), NCBI (https://www.ncbi.nlm.nih.gov/) and the CTD (http://ctdbase.org/). “Oral Lichen Planus” was used as a key word.

#### Acquisition of the common targets of quercetin and OLP

The common targets were collected based on the Venny website (http://bioinformatics.psb.ugent.be/webtools/Venn/) by entering the selected drug targets and disease genes into the website.

#### Protein–protein interaction networks construction, topology analysis and MCODE cluster analysis

PPI network was constructed by entering the common targets of quercetin and OLP into the STRING database (https://string-db.org/cgi/input.pl). The PPI network was imported into Cystoscape 3.8.0. Topological analysis was performed using the cytohubba tool, and the hub nodes were set as the top ten nodes. The key genes were ranked by the following four aspects: maximum neighborhood component (MNC), density of maximum neighborhood component (DMNC), maximal clique centrality (MCC) and degree (Deg). On the other hand, the MCODE module was used to analyze gene clusters and screen core targets of each gene cluster.

#### Gene Ontology (GO) and Kyoto Encyclopedia of Genes and Genomes (KEGG) analysis

In this study, the common targets of quercetin and OLP were enriched by GO and KEGG enrichment analyses. Three aspects were included in the GO enrichment analysis: biological process (BP), molecular function (MF), and cell component (CC). All the GO and KEGG enrichment analysis results were selected by *p* values ≤ 0.05, and the data were collected using the String database. R 4.0.2 software was used to draw GO and KEGG graphics.

#### Construction of the drug–disease–pathway–target network

To understand the complex interactions between pharmacological components, diseases, and corresponding targets, a visual network was established using Cystoscape 3.8.0. It included corresponding components, therapeutic diseases, targets, and main signal pathways and directly revealed the characteristics of the multi-component and multi-target effects of drugs associated with the pathogenesis of the disease.

### In vivo experiments

#### Ethics statement

The present study was approved by the institutional review board of Nanjing Medical University (permission number 2014-132). The procedures were performed according to the guidelines of the Declaration of Helsinki. All study subjects provided written informed consent.

#### Patients and controls

66 patients with OLP and 33 healthy controls (males 18, females 15, age 44.80 ± 2.422) were included in the study. Healthy controls were classified into the normal group. According to the guidelines set forth by van der Meij et al.^49^, OLP patients were divided into two groups: erosive and non-erosive group. Each group consisted of 33 patients (males 17, females 16, age 43.50 ± 2.731; males 16, females 17, age 46.60 ± 2.580). The non-erosive group was defined in accordance with the Wickham striae on the oral mucosa with no painful, ulcerated or erythematous areas. The erosive group was principally characterized by painful, ulcerated, congested and erythematous areas. The healthy controls did not have oral mucosal diseases or autoimmune diseases and had not used antibiotics or immunologic agents for the past 3 months^50^. There were no significant differences in age or sex between the three groups (*p* > 0.05). They were recruited from the Department of Oral Medicine of the Affiliated Hospital of Stomatology at Nanjing Medical University from August 2020 to January 2021.

#### The isolation of peripheral blood T lymphocytes and the drug preparation

Before 10 a.m., 10 mL of venous blood was drawn from each individual and collected in EDTA-containing tubes. T lymphocytes were isolated from the peripheral blood using Lymphoprep™ separation and EasySepTM human CD3 positive selection kit (Stemcell Technologies, VAN, Canada). Blood was diluted with phosphate-buffered saline (PBS) (Gibco, Grand Island, NY, USA) at a 1:1 ratio. Then, 5 ml diluted blood was overlaid on 5 ml lymphocyte separation medium and centrifuged for 20 min at 800*g* (ACC:1 DEC:0). After centrifugation, the buffy coat was collected into a new tube and spun at 650*g* for 10 min. Human CD3 positive selection kit was used to isolate T lymphocytes. Finally, T lymphocytes were maintained in RPMI 1640 complete medium (Gibco, USA) supplemented with 10% FBS (Gibco, USA), 1% penicillin, and streptomycin in an incubator set at 37 °C and 5% CO_2_. Quercetin (Solarbio, Beijing) was dissolved in 100% dimethyl sulfoxide (DMSO; Sigma Aldrich, USA) to form a 1000 μmol/L stock solution before further dilution. According to the previous literature^51,52^, we selected several concentrations as follows: 0, 1, 5, 10, 20, 40, and 100 μM. ELISA assay and Annexin V/propidium iodide (PI) assay was conducted to detect the protein expression of IL-6 and IFN-γ and the apoptosis of T lymphocytes.

#### Cell purity identification

PE-labeled anti-human CD3 antibody (BD, New Jersey, USA) was used for cell purity identification. According to the ratio of 1:1 antibody/10^6^ cells, the cells were incubated and stained for 20 min at 25 °C in the dark. Finally, the resuspended cells were detected by flow cytometry (FACS-400, BD, New Jersey, USA) after washing twice with PBS. Cells without antibodies were used as negative controls.

#### Cell proliferation assay

Cells were seeded at a density of 5000 cells per 100 μL in a 96-well plate. After a 24 h incubation growth period at 37 °C, cells were treated with various concentrations of quercetin (0, 10, 20 and 40 μM). Then, 10 μL of Cell Counting Kit-8 (CCK-8, Beyotime, China) was added into each well at 0, 12, 24, 48, and 72 h. Incubation for 4 h at 37 °C was required, after which the optical density (OD) was measured at a wavelength of 450 nm using a microplate reader (Molecular Devices, California, USA). All concentrations were tested in triplicate, and the experiments were repeated three times. GraphPad Prism 6 software was used to generate growth curves.

#### Cell apoptosis assay

Annexin V/propidium iodide (PI) assay kit (BD, New Jersey, USA) was used to evaluate the apoptosis of T lymphocytes. T lymphocytes were seeded into four groups as described previously before in 24-well plates. After washing twice with PBS, the T lymphocytes were resuspended in the binding buffer and stained with Annexin V-FITC and PI for 15 min in the dark. A flow cytometer (FACS-400, BD, New Jersey, USA) was used to analyze the results. The sum of the percentage of Annexin V (+)/PI (−) cells and Annexin V (+)/PI (+) cells represents the rate of apoptosis.

#### Cell migration assay

The 24-well Transwell plates with 4.0 µm pore polycarbonate membrane inserts (Millipore, Massachusetts, USA) were used to detect the effect of quercetin on the migration of T lymphocytes. In summary, 1 × 10^5^ of T lymphocytes were resuspended in 200 μL serum-free RPMI 1640 medium and plated on the upper side of the filter, while 800 μL complete medium with different concentrations of quercetin (0, 10, 20, and 40 μM) were placed in the lower plate. After incubation at 37 °C in 5% CO_2_ for 24 h, non-migrating cells on the upper surface of the membrane were gently removed with a cotton swab. Cells were stained with 4% paraformaldehyde (Sigma Aldrich, USA) to fix the migrated cells in the lower chamber for 20 min, and the cells were stained with 0.25% crystal violet (Beyotime, China) for 15 min at 25 ℃. Next, a microscope (Olympus, Japan) was employed to capture images of the cells that migrated to the lower chamber. Five high-powered fields per filter were randomly selected under a microscope to count the number of migrated cells. The cell migration rate was calculated as follows: (Mobility) = (total number of migrated cells in the treatment group − total number of migrated cells in the control group)/total number of migrated cells in the control group.

##### ELISA

T lymphocytes were treated with quercetin for 24 h at varying concentrations (0, 10, 20, and 40 μM) in 24-well plates. The culture supernatant was collected and centrifuged at 1200*g* for 20 min. Finally, the corresponding ELISA kits (Valukine, Minnesota, USA) were used to detect the protein expression of IL-6 and IFN-γ according to the manufacturer’s instructions. Each sample was assessed in triplicate.

#### Statistical analysis

All statistical analyses were performed using the Statistical Package for Social Sciences version 17.0 software (SPSS) and the computer-assisted GraphPad Prism program (Prism version 5.0, GraphPad Software, San Diego, CA). Data are presented as mean values ± standard error of the mean (SEM). Statistical comparisons were performed using one-way analysis of variance (ANOVA). Statistically significant differences were accepted when the *p* value was ≤ 0.05.

## Supplementary Information

Supplementary Information.

## Data Availability

The datasets generated and analyzed during the current study are available from the corresponding author on reasonable request.
