# Supplementary material for: Reveals of quercetin’s therapeutic effects on oral lichen planus based on network pharmacology approach and experimental validation
Source: Sci Rep. 2022 Jan 21;12:1162. doi: 10.1038/s41598-022-04769-z (PMC8782947; doi:10.1038/s41598-022-04769-z)

Supplementary Material

Title page

Title: Reveals of quercetin's therapeutic effects on oral lichen planus based on network pharmacology approach and experimental validation

Authors: Zhibai Zhao[1,4#], Linglin Wang[1,2,3#], Mengna Zhang[1,2,3], Chenyu Zhou[1,2,3], Yanting Wang[1,2,3], Jiangmin Ma[5**] and Yuan Fan[1,2,3*]

Author affiliations:

1. Department of Oral Mucosal Diseases, The Affiliated Stomatological Hospital of Nanjing Medical University, Nanjing, China
2. Jiangsu Province Key Laboratory of Oral Diseases, Nanjing, China
3. Jiangsu Province Engineering Research Center of Stomatological Translational Medicine, Nanjing, China
4. Department of General Dentistry, Stomatological Hospital of Xi'an Jiao Tong University, Xi'an, Shanxi, 710004, China
5. Department of Stomatology, Kunshan Hospital of Traditional Chinese Medicine, 189 Chaoyang Road, Kunshan , China

# These authors contributed equally: Zhibai Zhao and Linglin Wang.

Corresponding authors:

** The second corresponding author: Jiangmin Ma, Department of Stomatology, Kunshan Hospital of Traditional Chinese Medicine, 189 Chaoyang Road, Kunshan, Jiangsu, 215300, China. majiangmin920@163.com.

* The first corresponding author: Yuan Fan, Department of Oral Mucosal Diseases, The Affiliated Stomatological Hospital of Nanjing Medical University, 136 Hanzhong Road, Nanjing, 210000, China. fanyuan@njmu.edu.cn, ORCID ID https://orcid.org/0000-0002-4759-5625, +86-13913910018

1. **Supplementary Table. S1**

The effect of different concentrations of quercetin on the expression of IL-6 of T lymphocytes. After the incubation with different concentrations of quercetin for 24 h. The 10, 20, 40, and 100 μM quercetin induced a lower IL-6 protein level than the control (** $p < 0.01$, *** $p < 0.001$ versus the control group, n = 3). There was no obvious difference between the 40 and 100 μM quercetin group($p > 0.05$).

concentrations of quercetin (μM)	concentration of IL-6 (pg/ml)
control	145.34±3.77
1	144.49±10.18
5	139.53±4.39
10	76.98±2.56 **
20	42.17±3.10 ***
40	28.00±0.86 ***
100	27.36±2.31 ***

2. **Supplementary Table. S2**

The effect of different concentrations of quercetin on the expression of IFN-γ of T lymphocytes. After the incubation with quercetin for 24 h. The 10, 20, 40, and 100 μM quercetin induced a higher IFN-γ protein level than the control (** $p < 0.01$, *** $p < 0.001$ versus the control group, n = 3). There was no obvious difference between the 40 and 100 μM quercetin group($p > 0.05$).

concentrations of quercetin (μM)	concentration of IFN-γ (pg/ml)
control	46.57±5.79
1	48.24±5.42
5	52.72±5.45
10	97.02±9.02 **
20	171.16±18.72 ***
40	301.71±17.48 ***

100	308.69±25.02 ***

3. Supplementary Fig. S1

The effect of different concentrations of quercetin on the apoptosis of T lymphocytes. The percentage of apoptosis was composed of early and late apoptotic rate. The rate of Annexin V-FITC (+)/PI (-) represents the early apoptotic rate. The rate of Annexin V-FITC (+)/PI (+) represents the late apoptotic rate. The 20, 40, and 100μM quercetin group had a high apoptosis rate. (* $p < 0.05$, *** $p < 0.001$ versus the control group, n = 3)

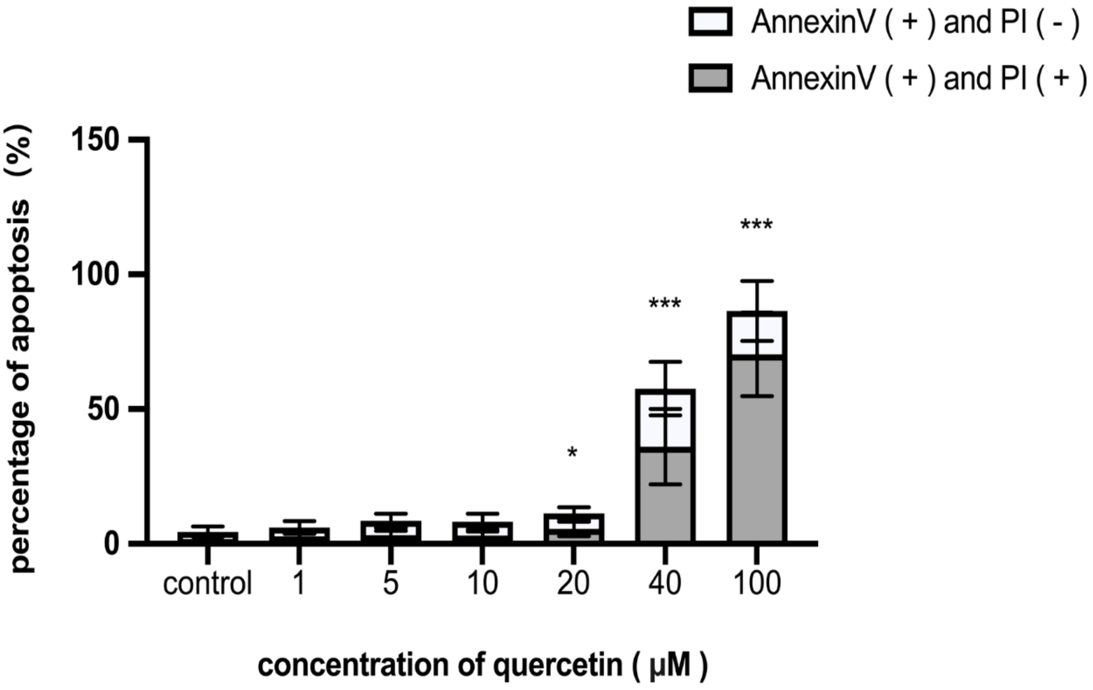

Supplement: Supplementary file 1 — Supplementary Information. [file 41598_2022_4769_MOESM1_ESM.pdf]
